# Community and Health Care Provider Perspectives on Barriers to and Enablers of Family Planning Use in Rural Sindh, Pakistan: Qualitative Exploratory Study

**DOI:** 10.2196/43494

**Published:** 2023-03-10

**Authors:** Zahid Ali Memon, Abeer Mian, Sophie Reale, Rachael Spencer, Zulfiqar Bhutta, Hora Soltani

**Affiliations:** 1 Centre of Excellence in Women and Child health Aga Khan University Karachi Pakistan; 2 Department of Allied Health Professionals, College of Health, Wellbeing & Life Sciences Sheffield Hallam University Sheffield United Kingdom; 3 Department of Nursing and Midwifery Sheffield Hallam University Sheffield United Kingdom

**Keywords:** gender, sexual and reproductive health, modern contraception, family planning

## Abstract

**Background:**

Unmet need for family planning in Pakistan is high, with 17% of all married women wanting to avoid or delay pregnancy. However, they cannot owing to a lack of access to modern contraception and sociocultural hindrances. With the modern contraceptive prevalence rate stagnant at approximately 25% over the last 5 years, it is important to explore barriers and enablers to modern contraception uptake to reduce maternal and child mortality and improve reproductive health outcomes for young girls and women.

**Objective:**

A formative research approach was taken to explore community member and health care provider perspectives on access to and use of family planning methods in 2 rural districts of Sindh, Pakistan. The broader goal of this study was to provide evidence to design and implement a socioculturally appropriate family planning intervention within the existing service delivery platforms to increase modern contraceptive uptake in the context of rural Sindh.

**Methods:**

A qualitative exploratory design was used. Between October 2020 and December 2020, 11 focus group discussions and 11 in-depth interviews were conducted. Focus group discussions were held with men and women from the community, including adolescents, to build an understanding of community beliefs and concepts regarding modern contraceptive methods. In-depth interviews were conducted with health care workers and explored intersections between family planning and reproductive health service delivery at the facility and outreach levels.

**Results:**

The findings revealed that limited financial autonomy, restricted women’s mobility, discriminatory gender norms, and cultural practices left women with little opportunity for independent decision-making on the use of modern contraceptive methods. Furthermore, facility-level and supply-side barriers, including frequent stock-outs of modern contraceptives combined with a lack of capacity of health workers to provide quality family planning services and counseling, played an important role in demotivating women from seeking services. In addition, a lack of integration of family planning with maternal and child health service delivery at the health system level was emphasized as a major missed opportunity for contraceptive uptake. Several demand-side barriers to family planning uptake were also highlighted. These included husbands’ or in-laws’ disapproval, social stigma, and perceived fear of side effects regarding modern family planning method use. More importantly, a lack of adolescent-friendly reproductive health services and spaces for counseling was identified as a critical intervention area.

**Conclusions:**

This study provides qualitative evidence on issues related to the effectiveness of family planning interventions, specifically in the context of rural Sindh. The findings emphasize the need to design socioculturally appropriate and health system–relevant family planning interventions—the effectiveness of which can be improved through their integration with maternal and child health service delivery mechanisms, consistent service provision, and opportunities for the capacity building of the health care workforce.

**International Registered Report Identifier (IRRID):**

RR2-10.2196/35291

## Introduction

### Background

Over the past 4 decades, family planning (FP) has proven to be an effective intervention in the aversion of maternal and child mortality [1] as well as poverty reduction [2]. The use of FP methods has been associated with a 32% reduction in the likelihood of maternal mortality and a 10% reduction in child mortality [2]. There is evidence from the global literature on FP further revealing that 20% of obstetric-related and 90% of abortion-based deaths could be prevented through the use of modern contraceptive methods by women desiring to delay or cease having more children [2]. By preventing unintended or undesired pregnancies, FP enables women to have freedom of decision regarding parity, pregnancy spacing, and their sexual health. This evidence presents FP as an important health reform (not only for maternal and child health but also for sexual and reproductive health) with high cost-effectiveness [3], specifically for use in the context of low- and middle-income regions, including Africa and South Asia. In addition, by increasing modern contraceptive uptake and prevalence from 10% to 60%, FP programs implemented at both the facility and community levels have played an important role in fertility reduction from 6 births to almost 3 per woman in the context of low- to middle-income countries [2].

Many recent studies have undertaken robust impact evaluations of FP programs, specifically at the community level. These studies have established that the disruption and discontinuation of FP services lead to a considerable reduction in modern contraceptive use and continuity. This, in turn, leads to the deterioration of sexual and reproductive health outcomes, marked by increased unintended pregnancies, maternal complications, and abortion rates among adolescent girls and women of reproductive age, as was shown to be the case in both Ghana and Bangladesh [4].

Modern contraception also substantially contributes to the realization of the human rights agenda. This agenda is premised upon achievements in universal primary schooling for women as well as women’s empowerment through improved sexual and reproductive health, educational, economic, and social outcomes. In addition, FP is considered a crucial contributor to environmental well-being and sustainability through its ability to reduce both the potential for global environmental degradation and the demand for water [2].

When put together, these achievements in education, empowerment, and the environment enabled by FP render it a highly effective and feasible tool in progress towards the fulfillment of the Sustainable Development Goals—specifically, goals related to poverty reduction, health and well-being, and gender equality [5].

Despite these gains, low- and middle-income countries continue to engage in poor contraceptive practices combined with uncontrolled population growth and an ever-rising unmet need for FP. This is despite the implementation of government-mandated FP and contraceptive service programs and interventions being rolled out at the local, regional, and national levels. Although many of these countries have FP policies in place, the literature claims that inadequate funding and lacking political will have rendered their implementation ineffective [6]. Several studies in the literature further equate poor performance on FP indicators and low modern contraceptive prevalence rates with a lack of socioculturally appropriate approaches to FP programs. An additional factor emphasized in the literature is the low-skilled health care workforce, specifically in terms of poor standards of FP, contraceptive counseling, and service delivery in low-income countries [7].

A more recent study conducted on contraceptive decision-making in low- and middle-income countries also found that a lack of men’s participation in FP programs and policies proved to be an important barrier to women’s reproductive rights—in terms of their ability and freedom of choice to adopt a modern contraceptive method. This also reduced women’s likelihood of continued appropriate use, especially if it was against the will of their partners [8].

A prime example of such a country where a combination of these factors has continually resulted in modern contraceptive use remaining exceptionally low is Pakistan. This is despite the efforts of the flagship Lady Health Worker Program, mandated in 1994, to provide FP services to families in their homes in underserved communities across the country. Pakistan is the fifth most populous country in the world, with 68% of its population aged <30 years [9]. Moreover, with a total fertility rate of 3.6, the country’s population is growing much faster than that of its neighboring countries in South Asia [9,10]. The modern contraceptive prevalence rates have been stagnant at approximately 25% during the last 5 years. Furthermore, the unmet need for FP is high, with 17% of all married women wanting to avoid or delay their pregnancies but being unable to do so because of inaccessibility to contraception methods [10].

Young married couples (aged 15-24 years) are at a greater disadvantage and face greater social pressures to bear children early in their married lives [11]. According to the latest national data, approximately 14% of adolescent girls (aged 15-19 years) are currently married, and approximately one-fifth of them have begun childbearing in Pakistan. Moreover, the proportion of married adolescent girls who want more children has increased in recent years (from 89% to 96%) [10]. Furthermore, unintended pregnancies have resulted in an estimated 2.2 million abortions (carried out predominantly by unskilled health care providers) every year in the country [12,13]. Therefore, addressing the unmet need and social and gender barriers to the use of FP services is crucial for empowering women and girls and also reducing maternal and child morbidity and mortality [14]. In line with this, studies in the local literature call for a need to further explore community perspectives, practices, and attitudes toward FP and modern contraceptive uptake. Lower literacy levels among women in Pakistan combined with low adoption of healthy timing and spacing of pregnancy practices demand an in-depth inquiry into barriers and enablers at the community level but, more importantly, at the health system level [15].

Thus, to address this unmet need and the low use of modern contraceptives, the role of health care workers and health management staff needs to be recognized as central in ensuring availability and quality of FP information, products, and services. Despite this, providers’ own perceptions, beliefs, experiences, and inputs are almost completely excluded when it comes to informing the design and delivery of interventions [16]. Thus, there is a need to design evidence-based interventions at the grassroots level to support both women and health care providers in identifying, understanding, and meeting reproductive goals, primarily through awareness and access to FP methods of choice [17].

### Objectives

Designing such evidence-based interventions requires the exploration of contextual factors through qualitative methodologies and field-level research to explore the barriers to and facilitators of FP uptake. However, scarce research exists on this topic in the context of rural Sindh, Pakistan. Therefore, this study aimed to explore perspectives of community members and health care providers on barriers and enablers in access to and use of FP and modern contraceptive methods in 2 rural districts of Sindh.

## Methods

### Study Design, Setting, and Participants

This qualitative study used an exploratory design to explore the barriers, facilitators, and perceived acceptability of FP services among communities and health care providers. The data collection methods used were in-depth interviews (IDIs) and focus group discussions (FGDs). IDIs served as an important tool to gain firsthand information and experiences regarding FP and sexual and reproductive health—both of which remain highly sensitive topics in the study community. IDIs were designed to explore intersections between FP and reproductive health related to the household and individual levels. FGDs were applied in cases where shared concepts and community-held belief systems regarding FP methods, use, and barriers to access needed to be understood [18].

Furthermore, FGDs allowed participants the opportunity to express their preconceived notions regarding FP methods, use, and their repercussions in all aspects, including the social, physical, and economic aspects. Thus, a combination of both methods allowed for the extraction of valuable insights from participants on topics that are relatively less researched [19].

The study was conducted in Sindh, which is the second most populous province in Pakistan. Sindh consists of 29 districts. This study was conducted in Matiari and Qamber Shahdadkot—2 rural and remote districts. The rural districts of Sindh are characterized by poor maternal and child health indicators and low use of FP products, methods, and services [15]. Therefore, their selection for this study was warranted because of their urgent need for FP services.

Participants included (1) health care providers or workers associated with the Department of Health, Government of Sindh, for >1 year; (2) married men and women; and (3) adolescent boys and girls. Those who refused to provide voluntary informed consent or did not agree to participate in the study were excluded.

A purposive sampling technique was used to select participants based on their relevance to the study objectives and their experiences of FP service delivery and uptake of FP and modern contraceptive methods. The sample size was determined through theoretical saturation.

### Development of the Topic Guide

The core research team, comprising the principal investigator and coinvestigators, developed the topic guides for the FGDs and IDIs. Open-ended guides were developed in English and included questions related to the research objectives. These questions were drafted based on the local literature on the topic of FP, modern contraception, and sexual and reproductive health. The purpose of the guides was to gather information regarding barriers to and facilitators and acceptability of FP and contraceptive services at the community level and details of services provided at the community and facility levels.

The topic guides were translated into the local Sindhi language and then modified following a pilot test at each field site. This included modifications to ensure ease of language and communication of sensitive concepts.

### Data Collection

IDIs and FGDs were conducted between October 2020 and December 2020. All the FGDs and IDIs were audio recorded after obtaining written and recorded consent from all study participants. The audio recordings of FGDs and IDIs were first transcribed in the local language (Sindhi) and then translated into English. The duration of each FGD was between 75 and 120 (mean 98, SD 9.87) minutes, whereas the duration of each IDI was 45 to 60 (mean 52.5, SD 7.24) minutes.

The team comprised a facilitator (who moderated the interview) and note taker. The team was selected based on previous experience and expertise in conducting qualitative research. Furthermore, a 2-day training workshop was conducted by the investigators. This training was focused on communicating specific objectives of the research, associated research methods, and providing the team with a basic fundamental understanding of FP and sexual and reproductive health concepts and practices.

### Data Analysis

The audio recordings of FGDs and IDIs were manually transcribed verbatim in Sindhi and then translated into English by a health professional. Following the transcription of the interviews, data were analyzed using inductive thematic analysis. Each transcript was reviewed by 2 independent reviewers. Both reviewers began by identifying initial codes to elicit the views of participants [20].

Axial coding was then applied to various concepts, quotes, and subthemes related to these initial codes. The refined categories were then grouped together, and similar categories were merged to develop themes. These codes were then shared between the independent reviewers to cross-check and triangulate all codes generated. As per this process, new codes were also added under the larger themes identified. Finally, selective coding was performed to establish the key themes and findings [21].

The final themes and interpretations were debriefed to the study participants to seek their perspectives and enhance the credibility of the study findings through a value check. For the most part, the study participants agreed with the study findings, with a slight difference of opinion between participants regarding the role of side effects associated with modern contraceptive methods. The difference emerged from community women and men emphasizing the role of side effects as one of the more important factors that discouraged potential users from adopting modern contraceptives. In contrast, health care providers underplayed the role of side effects and did not think that modern contraceptive methods had severe side effects.

### Ethics Approval

Ethics approval was obtained from the Ethical Review Committee of the Aga Khan University on July 16, 2020 (2020-3606-18261). The study protocol was approved by the National Bioethics Committee of Pakistan (4-87/NBC-514/22/857). In addition, ethics approval from Sheffield Hallam University (ER 41271675), United Kingdom, was received.

### Informed Consent

Researchers sought informed verbal consent from the participants after providing an explanation of the study objectives, procedure, and right to withdraw from the study at any time without consequence. Potential benefits and risks were also explained to study participants using lay terminology in the local Urdu language. Once participants agreed to join the study, a copy of the consent form was read to them, and they were given opportunities to ask questions and express concerns. Following this, the participants who showed willingness to take part were provided the consent form for signature or thumbprint and, as per protocol, were handed a copy of the consent form to keep. All participants were informed that taking part was completely voluntary and that they could choose not to participate in the study or withdraw at any time.

Moreover, all data collected from respondents as part of the FGD and IDI transcripts were anonymized through a specified code assigned to each individual for deidentification. This was done to ensure participant privacy and confidentiality.

## Results

### Overview

Overall, 11 FGDs and 11 IDIs were conducted. FGD respondents included men and women aged between 18 and 45 years as well as adolescent boys and girls (married and unmarried) aged between 14 and 19 years at the community level. IDI respondents included health workers such as the head of department, administrator from the Department of Health, gynecologists, medical officers, staff nurses, lady health visitors (LHVs), lady health workers (LHWs), community health workers, health visitors, and midwives. Participants at the community level reported having either no formal education or some level of primary education. At the health care provider level, all participants reported having secondary education at the intermediate, bachelor’s, and master’s levels, including Bachelor of Arts, Bachelor of Science, Bachelor of Science in Nursing, General Nursing, Master of Arts, or Master of Science. Most participants were aged between 10 and 19 years (41/74, 55%), with others aged between 20 and 57 years (33/74, 45%).

**Figure 1:**
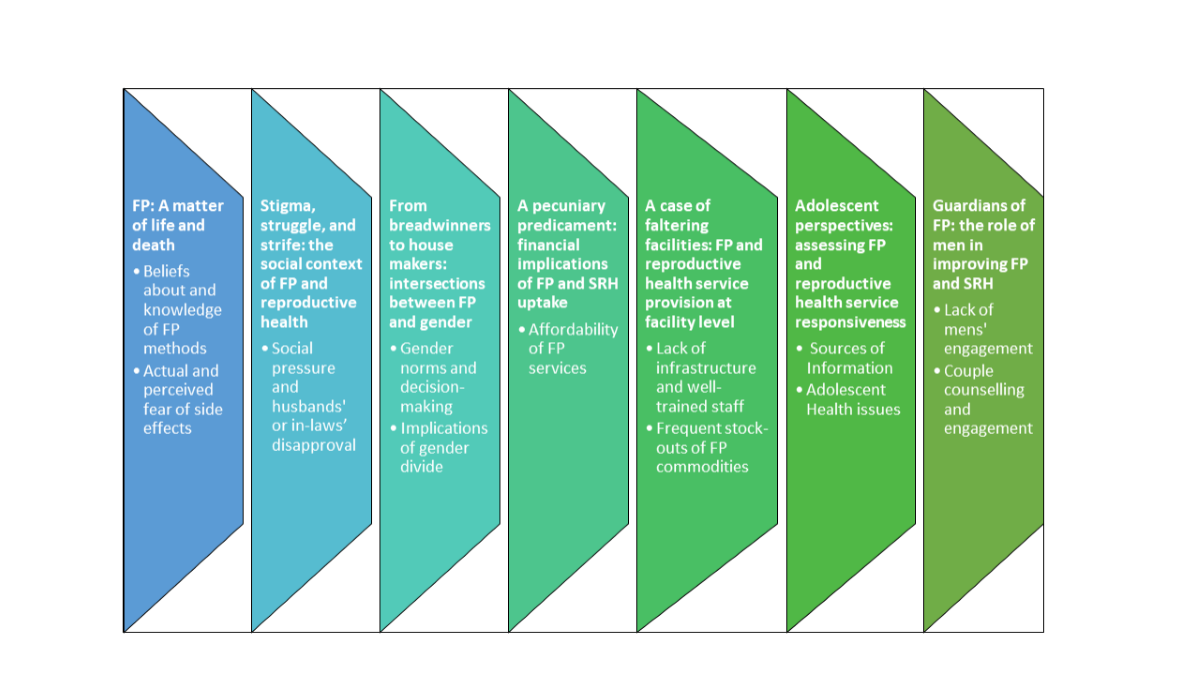
List of themes and subthemes. FP: family planning; SRH: sexual and reproductive health.

As part of the analysis, 7 major themes emerged as outlined in Figure 1. These were (1) FP: a matter of life and death; (2) stigma, struggle, and strife: the social context of FP and reproductive health; (3) from breadwinners to house makers: intersections between FP and gender; (4) a pecuniary predicament: financial implications of FP and sexual and reproductive health uptake; (5) a case of faltering facilities: FP and reproductive health service provision at the facility level; (6) adolescent perspectives: assessing FP and reproductive health service responsiveness; and (7) guardians of FP: the role of men in improving FP and reproductive health.

### Theme 1: FP—A Matter of Life and Death

#### Beliefs About and Knowledge of FP Methods

Several myths and misconceptions surround the nature and use of contraceptive methods at the community level, many of which are socioculturally concocted. During the group discussion, participants exhibited an overwhelmingly negative perception of modern FP methods (such as intrauterine contraceptive devices [IUCDs], implants, injections, and oral contraceptive pills) because of perceptions of side effects associated with them. These were expressed as irregular menstruation or bleeding, weight gain, stomach problems, infertility, or delayed fertility after discontinuation of the method.

Perceptions of the IUCD method specifically included them being considered “foreign matter” and chances that they would be “expelled” from the body at any time. Some also believed that implants caused infertility, as expressed in the following words:

Mostly women who come to us express their fear that FP methods cause severe bleeding and reduce blood in their bodies. They also fear that the IUCD can get displaced. Some women even believe that implants and pills cause infertility.

IDI, LHV

Some women, due to the influence of their neighbors, have come to believe that their uterus will stop functioning or they will face complications if they use an FP method.

IDI, gynecologist

Such misinformation regarding the use of contraceptive methods was also shared and expressed by some men, who believed that these methods presented a physical danger to the lives of their spouses. In this study, some went as far as claiming that they could be life-threatening and fatal. Efforts by local health care providers to address and rectify these perceptions held by some men through counseling and visitations at the household level were effective in some cases and ineffective in others. This was expressed by a health care provider based on their own experience:

Some men agree with their wives and allow FP whereas some men become angry with their wives. They get angry and then scold us workers, saying that their wives will lose their lives with this method and it is not good from an ethical point of view. We try to counsel them, some men understand our point of view whereas some men don’t.

IDI, LHV

In addition, most participants (46/74, 62%) highlighted the role of religion as an enabler for FP acceptance and uptake. They claimed that religion encourages the concept and practice of birth spacing and seeks to protect the well-being and health of a mother and child, as follows:

As far as religious barriers are concerned, people have very less knowledge regarding religion otherwise our religion is not that strict...We are allowed to have a duration of 2 years between having children. These are all fake myths that it is a sin and people will get punished, this is all due to lack of awareness.

IDI, gynecologist

In tandem with this view, it was further expressed that religion empowers women to be equal decision makers, specifically with regard to their reproductive rights, choices, and freedoms. In that regard, it emphasizes matters related to the health of women as matters that should involve joint decision-making and collective power shared between a man and woman. To this effect, the following was expressed:

The reason is that whatever our religion and society tell us, when a man and woman discuss and take a decision together, they should build their life together, they should be thought of as two wheels of a car moving together.

IDI, gynecologist

In contrast, very few participants (2/74, 3%) held the notion that using contraceptive methods was against religious beliefs. A health care worker expressed the following:

The reason for not using FP is that a few people consider it bad. They say it is a sin and it is a disease and if we practice it, we will get illnesses (as a side effect). We will not be able to produce children then.

IDI, community midwife; CMW

#### Actual and Perceived Fear of Side Effects

The recurrently mentioned side effects in the *sever*e category included infertility, cancer, birth deformities, and physical and mental disabilities. Regarding women who did use a contraceptive method, many expressed that the pain and side effects that they experienced were a factor that demotivated them to continue with sustained method use. This was evidenced by specific reasons for IUCD removal, as expressed by a participant:

IUCD caused a lot of pain so I preferred using injection.

FGD, married woman

Perceptions of side effects associated with different FP methods were somewhat mixed among participants and led to different method preferences. Although many contended with the use of the IUCD, others expressed concerns arising from the injection method, as follows:

I think IUCD and condoms are best for patients because there are usually no complications in both of them. With injections, patients have issues in their menstruation.

IDI, gynecologist

It is important to note that some participants (4/74, 5%) expressed reduced physical pleasure from using an FP method as an undesirable side effect as well. In that regard, men’s pleasure (which could be interrupted by the physical use of contraceptive methods) takes precedence and might lead men to refuse FP uptake:

Men perceive that the use of condoms, reduces pleasure and causes allergies and therefore they think that they should not use it.

IDI, CMW

It was also pointed out that potential side effects and associated financial costs for treatment for addressing them made the uptake of contraceptive methods less desirable. This was especially common in cases where men were unwilling or unable to cover the cost of such treatment as well as the indirect costs related to it. These were reported to include transport costs and the loss of daily earnings to take family members to visit a facility for treatment of side effects and keep having to make these visits for treatment follow-up.

Some men expressed reluctance to opt for any contraceptive method in the first place to prevent any onset of side effects and the perceived ordeal of treatment possibly associated with them. A married man said the following:

I have a fear of side effects and method suitability associated with FP products.

FGD, married man

### Theme 2: Stigma, Struggle, and Strife—The Social Context of FP and Reproductive Health

Social pressure and stigma attached to FP use and the embarrassment or fear of being caught using contraception serve as a major deterrent to women seeking, accepting, and using FP services. This resistance is mostly, as reported by participants, perpetuated by husbands and mothers-in-law, who harbor demands for a greater number of children and a larger family size. The physical burden of this falls on the shoulders of women without any regard for their choice in the matter.

This forces women to seek contraceptive methods in a manner that is discreet or unknown to their disapproving men family members or in-laws. The key motivation for continuing to hide their use of FP methods stems from their fear of being blamed and the shame borne upon them for not giving birth to more children, as desired by their families:

Men don’t agree with FP or spacing between children. They prefer large family size and [women] keep it secret from their husband.

FGD, married woman

Many women use FP services secretly without the knowledge of their families. They don’t even come here (at the health facility) for fear of being found out. Therefore, these women ask health workers [LHWs] for the FP supplies, and they provide the supplies to women in the community, at their doorsteps.

IDI, LHV

In many cases, a woman’s worth and value in her household is dictated not only by her ability to give birth but also by parity—that is, the number of children she gives birth to and rears. This, in turn, dictates both her position and power in the household. Having fewer children, as reported by participants, is associated with the lowered worth of a woman, specifically in her roles as a wife, daughter-in-law, and homemaker. This subsequently lessens the importance given to her health and well-being within the household. A participant expressed the following:

They [women] think that if they stop having children or have less children, they lose their importance.

IDI, gynecologist

In addition to women having to face the disapproval of their household members, some are also subjected to verbal and psychological humiliation or even physical abuse for not giving birth to the desired number of children, as observed by a married woman herself:

A woman will seek permission from her husband before spending money on FP. It is the husband who is paying for the service. If she does not get the permission, she will be threatened and abused.

FGD, married woman

In contrast, it was also expressed (by very few participants; 11/74, 15%) that mothers-in-law have an empowering role to play in FP uptake for their daughters-in-law in that, in some cases, they will support their daughters-in-law to use FP methods and enable them to access these methods in a discrete manner, hidden from their own sons. Therefore, the matriarch of the household has a crucial role to play in the uptake of FP methods at the household level, whether at the level of resistance or of empowerment:

Sometimes the mother-in-law herself asks us for FP services for her daughter-in-law in private because her son doesn’t agree to it, so we can give her injections, tablets and condoms in private.

FGD, health care provider

### Theme 3: From Breadwinners to House Makers—Intersections Between FP and Gender

#### Gender Norms and Decision-making

Societal norms define men’s role as breadwinners, financial providers, and heads of household, whereas women are expected to stay indoors, do household chores, and serve as caregivers. By way of these norms, women are restricted to the household, managing day-to-day tasks such as cooking, cleaning, serving their in-laws, and catering to all needs of the household and its various members. These tasks demand constant physical labor from women, who are expected, by way of predefined gender roles, to contribute this labor to the household without any expectations in return, as told in the words of a young adolescent boy:

It is the responsibility of the woman to prepare food for the family and clean the house.

FGD, adolescent boy

As per these conventional roles, men inherit the reins of decision-making as heads of the household. These decisions vary mainly across expenditures related to health, education, and mobility of mothers and children within the household, as narrated as follows:

Women face many challenges. If the husband forbids his wife from using FP or does not give her money to avail the services, she cannot do it on her own. The man is the financial supporter. A woman looks to her partner for all her financial needs. If a husband does not practice FP and does not provide support to his wife, then the wife is helpless.

IDI, CMW

With men’s traditional “breadwinner” role tipping the decision-making dynamic in their favor, women and young girls have little say in matters concerning their lives. In most cases, as claimed by participants, this leads to women being deprived of an education. A lack of basic education and literacy leaves women unaware of their health rights, needs, and issues. They are unable to identify or communicate issues that arise within their physical and mental health and, therefore, are unable to express a need for seeking timely care. This is a perpetual cycle that begins with young girls lacking education and knowledge of sexual and reproductive health and eventually becoming mothers to daughters who receive no education and continue to experience the same health complications, issues, and fate, as follows:

Pressure on girls stops them from approaching us and discussing their issues. And going to the hospital to see a doctor for a young girl is considered unusual. These are the hindrances that stop them...We don’t have a system of visiting houses here and there is no such system of educating young girls because our families are not educated.

IDI, gynecologist

#### Implications of Gender Divide

However, the repercussions of women’s lack of access to education, decided and driven by men, stretch further beyond the domains of their health and well-being, affecting their agency to identify their health, economic, and social goals; explore choices related to them; and act upon them accordingly. In that regard, some men in the study described women as intellectually inferior and stated that they lacked problem-solving skills and were unable to make reasonable life decisions. In addition to rendering women incapable of making practical decisions, some participants also argued that it was by norm that the place of women in the community was to stay silent during all times when decisions were being made. This deprives women of their agency and an opportunity to provide any input in matters concerning functions of the household, as narrated by men participating in the FGD:

Women have low decision-making power due to insufficient problem-solving skills.

FGD, married man

Women should do their household work and stay silent when decisions are being taken.

FGD, adolescent boy

### Theme 4: A Pecuniary Predicament—Financial Implications of FP and Sexual and Reproductive Health Service Uptake

Affordability, including the cost of contraceptive methods and FP services as well as the cost of treatment for related side effects, was highlighted as a key barrier to accessing FP services. For men, these costs can be perceived as a financial loss or burden on already limited resources in lower-income households. In this community, where most households live below the poverty line, access to FP and sexual and reproductive health services can constitute a catastrophic expenditure. In that respect, the use of FP methods requires payments to purchase contraceptive products (in private-sector facilities) and having to visit facilities regularly for follow-up visits. This can financially strain families:

Sometimes we even counsel men if their wives have some sort of complication. We ask them to refer their wife to some other hospital but they deny this suggestion saying that they won’t leave. So they are not willing to make this effort. Some are genuinely poor...so poverty also plays an important role in our society.

IDI, gynecologist

For the most part, FP services are provided free of cost at government health facilities compared with the private-sector setup. Despite this, most participants (38/74, 51%) reported availing private-sector services for 2 reasons. First, FP and sexual and reproductive health products and services are more readily and consistently available in the private sector as compared with the public sector. Having access to uninterrupted services and commodities is essential for ensuring both the continuity and efficacy of FP method use. The second reason involved the perceived quality of services provided relative to both sectors. To this effect, it was observed throughout the interviewing process that many participants, including adolescents and married women, harbored a strong sense of mistrust of government services, specifically medicines provided by them, as follows:

Some people tell us that our medicines have less efficacy, and they are not useful because they are from the government.

IDI, LHW

### Theme 5: A Case of Faltering Facilities—FP and Reproductive Health Service Provision at the Facility Level

#### Lack of Adequate Infrastructure and Well-Trained Staff

In cases where women do access contraceptive services in either sector, issues arise at the health care provider level. Many participants (44/74, 60%) expressed during discussions that health care providers within their communities were not responsive to their FP needs. They further believed that these providers had poor knowledge and lacked skills related specifically to FP counseling and method use technicalities, as envisioned by a participant:

Counsellors [health staff] working in hospitals should have knowledge of health-related fields so that they have more knowledge and awareness on how to counsel people because these people come to the hospital on their own expense, so they want good service. We don’t have that level of knowledge to share.

FGD, adolescent

In the view of some participants, providers’ low competence and capability to deliver high-quality FP services is accredited to a lack of necessary training and capacity-building opportunities. Trainings, including refresher sessions for all providers (ie, facility- and community-level providers) on FP counseling, updated guidelines, and technical aspects such as surgical insertion and treatment of complications, are not regularly provided. This was identified as a key challenge for providers, as told by a participant:

Proper trainings are not given to staff on how to counsel people and how to emphasize our point of view. We should have certain measures and resources that people can avail in their communities for counselling.

IDI, gynecologist

Even the counsellors need guidance and training. They need comprehensive training on counselling.

IDI, gynecologist

Health care provider performance is further hindered by a lack of private and inclusive spaces at the facility level to provide detailed and confidential counseling to women and couples regarding sensitive issues of FP and contraception. The need for such spaces is specifically pertinent in cases where women choose not to disclose the use of FP methods to their family members:

There is so much rush in government hospitals. There is also no time or space available to explain in detail, information about services at government hospitals. Also, there are many patients and men around. You cannot explain it there.

IDI, CMW

I think the knowledge and behavior of healthcare providers is important to counsel clients in a very polite and practical way. Moreover, there should be a separate room for counselling.

FGD, married woman

However, even assuming that there were appropriate spaces developed for counseling, the core issue of overburdened staff still remains. Several unfilled vacancies coupled with the low availability of highly skilled, well-trained, and adequately qualified providers in the study districts translate into staff shortages. This not only hampers the provision of quality FP counseling and services but also lowers the importance given to FP over other mainstream facility functions such as outpatient department and emergency services, as described by a gynecologist:

We are overburdened in hospitals. There is shortage of time. One counsellor should be in the labour room as well, who can tell them [women] about antenatal care, delivery, breastfeeding, FP after delivery. There should be one dedicated counsellor who can separately provide counselling to each client.

IDI, gynecologist

In the secondary care hospital, there are no such counsellors available to facilitate patients. We do not have adequate human resources.

IDI, head of department

We have fewer staff and lack of space also hampers services. There should be separate spaces for different services along with adequate staff. There should be separate areas for waiting, record keeping, OPD, FP with separate staff. Besides, there is no security and privacy. Anyone can enter the hospital and we cannot stop them.

IDI, CMW

#### Frequent Stock-Outs of FP Commodities

Issues in the provision of FP services are further complicated by supply-side issues, most commonly stock-outs of essential FP supplies and commodities at both the facility and community levels. Health care providers emphasized the adverse impact of the unavailability of the method of choice on clients and their uptake of FP services:

We face shortages of contraceptives sometimes. If a patient is willing to take IUCDs and not willing to take pills and injections, then we must provide them that service. But due to unavailability of specific methods of choice, service provision is delayed and most of the time, those clients do not return to us.

IDI, gynecologist

Not only does this lead to frequent method discontinuation, but it also results in the gradual development of resistance to FP and perceptions of its inefficacy as a method to avert pregnancy. In that regard, interrupted access and use of FP methods as prescribed renders FP products useless and, more specifically, a waste of money and time—both of which are precious resources for the survival of low-income households in the study areas:

Unavailability of desired FP methods harms services. If the client returns without services, she may not come back. Her husband might change his mind, she might change her mind. Or she may not have the money to travel [again].

IDI, gynecologist

#### Missed Opportunities: Exploring an Integrated Model of FP—Maternal, Newborn, and Child Health Service Delivery

In light of all the aforementioned challenges expressed by participants, a strong recommendation that emerged specifically from health care providers was the need to integrate FP services into different delivery points at the facility level. They explained that integrating FP with the provision of maternal and child health services ranging from antenatal and postnatal care and pediatric services to immunization and outpatient department services would lead to reducing missed opportunities for sharing information and referring potential clients for contraceptive uptake. In that regard, women visiting these service delivery sites could be identified and referred for FP counseling and service uptake by health care providers, as expressed by a participant:

Many women approach health facilities for EPI or vaccination purposes. We should consider this an opportunity to spread awareness about FP.

IDI, head of department

Most women health care providers in this study (10/16, 63%) were in favor of engaging all service delivery platforms for the promotion of FP. This would allow for greater efficiency in identifying, referring, and counseling clients on the uptake of FP services among their catchment populations. The emphasis was put on the importance of providing FP counseling and information on the methods. To this effect, a community-based health care worker further emphasized the following:

Counseling is very important. And it is effective as well. Women, in particular, should be counselled on FP as soon as they come in for antenatal care. And if they are counselled well, women can remember to use the FP method after delivery. Therefore, it is very important that counselling should be done during antenatal care visits.

IDI, CMW

### Theme 6: Adolescent Perspectives—Assessing FP and Reproductive Health Service Responsiveness

The study findings further reveal that the absence of adolescent- and youth-friendly services at the facility level and adolescent engagement activities at the community level hinder the promotion of awareness, knowledge, and acceptance of contraceptive methods and uptake. Social stigma, shyness, and shame coupled with lack of awareness of FP information and services further restrict adolescents’ ability to avail reproductive health services. Thus, adolescents constitute a high-risk population for unwanted pregnancies and miscarriages and, therefore, qualify as priority target groups for FP and sexual and reproductive health interventions:

The adolescents and youth lack knowledge about FP and sexual and reproductive health and therefore there are many unwanted pregnancies and miscarriages.

IDI, LHV

Young people here are very shy. They do not consult their parents regarding their sexual and reproductive health. So why would they come to us? If a woman wants to take her daughter to a hospital due to some issue, then her neighbors would ask questions and stop her from visiting a hospital. Then they end up going for homeopathic treatment and therefore patients do not come to us.

IDI, gynecologist

I have not seen a single unmarried youth coming to this health facility for information or services related to FP or sexual and reproductive health.

IDI, LHV

Adolescents, both girls and boys, highlighted the lack of adolescent-friendly health facilities and community spaces where they could openly and confidently talk about their health issues. The health care providers and community health workers emphasized the need to engage adolescents and youth in creative FP and sexual and reproductive health programs:

We are young girls, and we face some sexual health problems which we cannot openly discuss even with our parents, because we feel shame and shy.

FGD, adolescent girl

If youth are educated, they will be safe and healthy. And they will not get involved in the wrong practices. If they lack awareness, someone will take them on the wrong path.

FGD, adolescent boy

We should educate young couples about FP and reproductive health because eventually they will be parents soon.

IDI, LHW

### Theme 7: Guardians of FP—The Role of Men in Improving FP and Reproductive Health

#### Lack of Men’s Engagement

An important finding emerging from this research was the role of men as positive influencers for FP and sexual and reproductive health service uptake. This is through their role in encouraging the use of contraception by their wives, sisters, and other family members at the household level, as well as advocating for FP service use among their peers. Outreach workers (men) can also engage social influencers and gatekeepers to increase sociocultural acceptance of contraceptive methods and gradually help remove barriers to access at the community level:

Engaging men is very crucial. Spouses can facilitate each other. There is a need to spread awareness, especially within the men.

IDI, head of department

Men should be involved in FP. It is their obligation. It is not only a woman’s responsibility. A man should know if he will be able to take care of his child properly. It is his responsibility too.

IDI, LHV

Despite the acknowledgment of men being urgently and closely engaged with FP, the culture of the community tends to remain, at large, ignorant of reproductive health needs, particularly of young boys. On the basis of participants’ responses, the exclusion of younger men and boys was an issue from the start. On the topic of training undertaken by LHWs for addressing men’s health issues, the following response was recorded:

Some trainings are done and some are in process, some girls [LHWs] have already completed the training but the number of trainings should be increased so that we have more knowledge and know what to say and do, when we are visiting people in the field.

IDI, LHW

Another element of this involves a lack of sufficient support for boys when they are growing up, specifically by way of being able to access information or knowledge regarding their health. It was revealed by adolescent boys that their trusted source of information regarding their health was their elders. More importantly, they expressed a sense of distrust toward physicians as failing to listen to their concerns and provide a suitable way forward for care or treatment. A young adolescent boy said the following:

There are some good physicians. Some listen and others do not. We want to know about our physical health. If there is a good physician, or a good man, who does not cheat us or does not bully us, we can consult him.

FGD, adolescent

Despite these realities, young boys expressed a demand for discussion groups, community-level platforms, and inclusive spaces where they can come together to discuss their issues. Specific issues that they identified for discussion during the course of FGDs and IDIs included (1) knowledge of sexual health, (2) beliefs about how FP and sexual and reproductive health services should operate, (3) sources of social support for sexual health (ie, access to sources of information), and (4) access to spaces that provide physical opportunities to seek care for sexual health issues. They further expressed the following:

We must have good physicians and good institutions from where we can get proper information. When we reach puberty, they should give us information about it… We should be well versed with sexual and reproductive health. We must be told about this.

FGD, adolescent

#### Couple Counselling and Engagement

In addition to a lack of men’s engagement, many participants (33/74, 45%) pointed out that there is no system for couple counseling on FP and sexual and reproductive health. Many claimed that, because of the absence of this, men are still not purposefully engaged in FP and sexual and reproductive health service use, and women often resort to having to take FP methods in a discrete manner by hiding it.

In cases where husbands know of FP use, they are not aware of the need for continued contraception and the need to access FP services at regular intervals to ensure the efficacy of the method. This leads to women being unable to follow-up at the clinics they receive services from as their partners do not understand the importance of taking them there for regular FP and reproductive health checkups. Gynecologists recalled the following:

It has also happened that women ask for IUCD insertions and request us not to tell their husbands. We advise them to first tell their husbands because this will affect their life in the future. Their husband may get to know about something, and it is better that the matter is sorted before that. So, the husband should be convinced from the start.

IDI, gynecologist

The couple should be involved in counselling. If one member of the couple is not understanding, then eventually the other member must be explained in a manner that he or she can be convinced.

IDI, gynecologist

## Discussion

### Principal Findings

This study is one of the first to explore the barriers to and facilitators and acceptability of FP and sexual and reproductive health service uptake in light of their intersection and interactions with existing health systems and service delivery platforms. Overall, these findings reveal that limited financial autonomy and decision-making power, restricted women’s mobility, discriminatory gender norms, and cultural practices where women are considered dependent and inferior beings left women with little opportunities for independent decision-making regarding their sexual and reproductive health and well-being. On the basis of the findings of this study, it is mostly the man who decides whether and when a woman can seek antenatal, delivery-related, postnatal, and postabortion care and if they can opt for FP services. Previous research from Pakistan has demonstrated the vital role of husbands’ attitudes and resistance as a prominent barrier to FP uptake [22]. Despite the dominant role men played in decision-making, a lack of men’s engagement in FP and absence of couple-centered FP counseling left women powerless and diminished their ability to make informed sexual and reproductive health decisions.

Over the last couple of decades, research has highlighted the role of gender norms and how they influence couples’ intentions and decision-making with regard to FP and reproductive health [23]. Many study participants (52/74, 70%) repeatedly suggested the need to engage men in FP initiatives. Previous research studies have also demonstrated that couples’ joint decision-making is a stronger positive determinant of contraceptive use and called for educating women and their husbands on FP and effective contraceptive methods [24].

This study highlighted several demand-side barriers to FP uptake, such as husbands’ or in-laws’ disapproval, social stigma, actual and perceived fear of side effects, and rumors about modern FP method use. Inconsistencies and unavailability of quality gender-responsive FP and sexual and reproductive health services were also emphasized by most participants (40/74, 54%) as a prominent barrier. The study findings further showed that poverty and cost of FP and sexual and reproductive health services led to low use. These findings are supported by previous qualitative research studies exploring FP practices and barriers to uptake that have underscored negative perceptions of FP methods, in-laws’ disapproval, religious concerns, and fear of side effects as important reasons for the low uptake of FP and modern contraceptive methods in Pakistan [25,26].

Moreover, the study findings also highlight supply-side factors, including frequent FP and essential medication stock-outs at the facility and community levels, understaffed health facilities, overstretched community health workers, and lack of investment in capacity building of staff members, as barriers to uptake. Previous research has highlighted the role of organizational factors such as supplies, equipment, infrastructure, workload, and larger social and community milieu in constraining providers’ ability to deliver quality services [27,28]. To this effect, improving communication and making FP and sexual and reproductive health services functional can revitalize uptake at the facility level [29].

In addition, most health care providers (9/16, 56%) emphasized the lack of integration of FP services with maternal, newborn, and child health (MNCH) services as a missed opportunity for increasing modern contraceptive uptake. In line with this, a systematic review exploring the impact of an integrated FP-MNCH model of service delivery found that it resulted in improved contraceptive uptake and was more socioculturally acceptable for community members. The addition of community-level elements involving FP counseling and service delivery at the household level was associated with greater uptake of modern contraceptive methods [30]. Similarly, a review of high-impact practices revealed that integrating FP services with routine immunization visits was a particularly effective way of increasing FP service coverage [31]. These findings are also consistent with World Health Organization recommendations for integrating FP referrals, counseling, and services with MNCH and immunization services [32]. The World Health Organization recommendations can be implemented at the facility level to target clients who come in contact with the health system for different health care needs. A review of research evidence sponsored by the US Agency for International Development for FP-MNCH integration demonstrated the feasibility of integrating MNCH and FP services across a variety of integration models, settings, and target populations [30]. Recent research evidence from Bangladesh has also advocated for systematically integrating postpartum FP into maternal and child health programs to improve birth spacing and prevent high-risk preterm births [33]. Thus, linking FP and postpartum FP counseling and referrals with routine immunization is considered a high-impact practice by many international organizations [31,34,35].

In addition, the findings also highlight the biased attitude of providers and unwelcoming behavior of physicians and nurses toward clients as important factors that demotivate women to access FP and sexual and reproductive health services. The findings underline the lack of understanding of health care providers about sexual and reproductive health issues, as well as gender issues in the community. This lack of understanding in providers’ knowledge negatively affects quality of care and service delivery [26].

Moreover, the findings reveal an almost absolute lack of adolescent-friendly sexual and reproductive health services. Social stigma and shame coupled with a lack of awareness and availability of sexual and reproductive health information and services for adolescents are a critical area for intervention. A recent landscape analysis report on adolescents in Pakistan also highlighted the lack of information about sexual and reproductive health and the dearth of avenues to openly discuss bodily changes, including puberty and menstruation [36]. This begins with a lack of adolescent-friendly services and spaces for boys in particular, which has reportedly led to the deterioration of their mental health as they remain unaware of their puberty, issues in their reproductive health, and when and how to seek help.

This ordeal for boys is worsened by participants’ claims that there is a lack of training and preparedness on the part of LHWs for supporting young boys. Youths in Pakistan further expressed their desire for sexual and reproductive health information and services to be provided in private to meet their needs [11]. This demand can be met through the initiation of community engagement activities using appropriate platforms that allow young boys to come together to identify and raise their questions and concerns regarding their reproductive health and rights, as well as those of girls, in a safe and inclusive space. These platforms should allow for interaction with health care workers who are trained in topics pertaining to adolescent health and education and who can further provide counseling to them regarding hygiene, puberty, early child marriages, gender equality, and FP. Counseling and educating men about these issues is a crucial first step in reducing sociocultural resistance toward FP and enabling men to support women to access FP and sexual and reproductive health services and protect their reproductive rights and freedom [11].

In contrast, knowledge of FP and sexual and reproductive health among adolescent girls is comparatively low, specifically within Gilgit-Baltistan, followed by Sindh and Balochistan. The use of any contraceptive method among young married couples aged 15 to 19 years is 7.4%, with only 5.9% using a modern method. Similarly, the use of any contraceptive method among young married couples aged 20 to 24 years is 18.3%, with only 13.4% using a modern method [10]. Limited access to quality FP and sexual and reproductive health services coupled with gender disparities and patriarchal sociocultural practices restrict women’s ability to make their own decisions about their health and access the desired services at the time and place of their choice. As a result, Pakistan ranks lowest on the Global Gender Gap Index at 153 out of 156 countries [37].

### Strengths

The strengths of this study include the richness of the data gathered from the qualitative inquiry. The qualitative approach was well suited to encouraging participants to voice their honest opinions, reservations, and concerns regarding contraception and FP, which is considered a taboo in the local context. In many ways, this helped develop an understanding of the causes behind resistance to FP despite dedicated efforts by the public and private sectors to provide these services at the community and facility levels.

Another important aspect of this research was its focus on capturing and including the perspectives and voices of adolescent girls on FP and sexual and reproductive health. These girls are usually left out of research spaces when it comes to reproductive health in the context of rural Pakistan even though they stand to benefit the most from awareness of and access to related services.

### Limitations

Alongside its strengths, this study has limitations. Owing to the qualitative nature of inquiry, the findings of this study regarding barriers to and facilitators and acceptability of FP and sexual and reproductive health services uptake cannot be generalized beyond the sample of participants to the larger population of women and girls in rural Sindh.

### Conclusions

This study uncovered themes exploring barriers to and enablers and acceptability of FP and sexual and reproductive health service uptake. In doing so, it sought to provide qualitative evidence on issues related to the effectiveness of FP interventions and programs, specifically in the context of Sindh. The findings of this study call for the need to design socioculturally appropriate FP interventions to be delivered consistently to the community by a trained and skilled health care workforce (that is provided with timely opportunities for capacity building).

At the health facility level, capacity building of health care staff; ensuring uninterrupted supply of FP commodities; and providing consistent gender-responsive, integrated, and client-centered services are recommended to improve FP uptake. At the community level, capacity building and training of community health workers, including LHWs, and increased men’s engagement have the potential to counter harmful gender norms, myths, and misconceptions regarding FP and sexual and reproductive health. Furthermore, developing adolescent-friendly spaces for girls and boys to discuss health-related issues serves as an important intervention window to promote awareness, acceptance, and uptake of FP and reproductive health services.

In conclusion, FP services could be improved by ensuring consistency in the provision of quality FP and sexual and reproductive health services at the facility and community levels by trained health care providers and workers. These services can be effectively provided through an integrated model of FP-MNCH service delivery that increases the efficiency, coverage, and sociocultural acceptability of FP methods and modern contraceptive uptake at the community level. The benefits of such integrated interventions have the potential to stretch beyond improved health outcomes for women to their education, economic and social empowerment.

## Abbreviations

CMW: community midwife
EPI: expanded program of immunization
FGD: focus group discussion
FP: family planning
IDI: in-depth interview
IUCD: intrauterine contraceptive device
LHV: lady health visitor
LHW: lady health worker
MNCH: maternal, newborn, and child health

## Data Availability

The data sets used for the paper and the study are available from the corresponding author upon request.
